# Timely referral to health centers for the prevention of cardiovascular diseases: IraPEN national program

**DOI:** 10.3389/fpubh.2023.1098312

**Published:** 2023-09-22

**Authors:** Fatemeh Hadavandsiri, Davood Khalili, Alireza Mahdavi, Mehdi Afkar, Afshin Ostovar, Seyed-Saeed Hashemi-Nazari, Somayeh Derakhshan

**Affiliations:** ^1^School of Public Health and Safety, Shahid Beheshti University of Medical Sciences, Tehran, Iran; ^2^Prevention of Metabolic Disorders Research Center, Research Institute for Endocrine Sciences, Shahid Beheshti University of Medical Sciences, Tehran, Iran; ^3^Center for Noncommunicable Disease Control and Prevention, Ministry of Health (MOH), Tehran, Iran; ^4^Community Medicine, Tehran Medical Sciences Islamic Azad University, Tehran, Iran; ^5^Osteoporosis Research Center, Endocrinology and Metabolism Clinical Sciences Institute, Tehran University of Medical Sciences, Tehran, Iran; ^6^Prevention of Cardiovascular Disease Research Center, School of Public Health and Safety, Shahid Beheshti University of Medical Sciences, Tehran, Iran

**Keywords:** compliance, WHO-PEN, IraPEN, relative factors, cardiovascular disease, risk

## Abstract

**Introduction:**

The IraPEN program is an adapted version of the WHO-PEN program designed to prevent four major non-communicable diseases in Iran. This study aimed to determine the rate of compliance and related factors among individuals participating in the IraPEN program for the prevention of cardiovascular disease.

**Method:**

In this study, compliance was defined as timely referral to the health center as scheduled, and the researchers approached four pilot sites of IraPEN from March 2016 to March 2018. Sex-stratified logistic regressions were applied to investigate factors related to compliance. However, it is important to note that in this study, compliance was defined as compliance to revisit, not compliance to taking prescribed medications or behavioral lifestyle changes.

**Results:**

The total compliance rate, including timely compliance and early and late compliance, was 16.5% in men and 23.3% in women. The study found that cardiovascular risk factors such as diabetes, hypertension, hypercholesterolemia, and being underweight were associated with lower compliance. The higher calculated risk of CVD was associated with higher compliance, but after adjusting for cardiovascular risk factors, high-risk individuals showed lower compliance. There was negligible interaction between sex and other factors for compliance.

**Conclusion:**

The compliance rate with scheduled programs for cardiovascular preventive strategies was very low, and high-risk individuals were less compliant, regardless of their high level of risk factors. The study recommends further training to increase awareness and knowledge regarding the IraPEN program and the prevention of non-communicable diseases among high-risk populations.

## 1. Introduction

Cardiovascular diseases (CVDs) are the leading cause of death worldwide, with approximately 80% of CVD deaths occurring in low- and middle-income countries, and approximately 40% of these deaths are labeled as premature (1). In Iran, the age-standardized rate of CVD mortality has slightly decreased from ~500 in 1990 to 400 per 100,000 in 2017, but it is still the leading cause of death. However, due to the aging of the population, the proportional mortality ratio has increased from 20% in 1980 to ~50% in 2017 (2). Iran ranks 10 in the Middle East and North Africa (MENA) region with a sociodemographic index (SDI) of 0.7, but its situation regarding high BMI, high fasting plasma glucose, high blood pressure, and high cholesterol is better than other countries, with an average rank of 5 among 21 countries in this region (3).

The WHO Package of Essential Noncommunicable Disease Interventions (WHO-PEN) is a set of cost-effective interventions developed for primary care in low-resource settings to detect and manage major non-communicable diseases, including CVD (4). IraPEN is an adapted revision of this program for primary healthcare settings in Iran, which has been integrated with the previous national programs for the control of non-communicable diseases, including hypertension and diabetes (5).

Treatment compliance is crucial to increase effectiveness and reduce healthcare costs in primary and secondary prevention (6–8). Non-compliance is associated with an increased risk of CVD, hospitalization, and higher healthcare costs (9, 10). Although there are no apparent predictors of compliance to revisit for cardiovascular diseases, some studies have mentioned that patients' attitudes, lifestyle preferences, education, support, and reminders appeared to have an association with compliance to medication (11–13). Additionally, some studies on other diseases have identified younger age, full-time work, short duration of illness, type of treatment, and psychological factors as possible risk factors for compliance (14, 15).

The IraPEN program was implemented as a pilot in four cities over 2 years (2016–2018). In this pilot, the 10-year risk of CVD was calculated using the regional 10-year World Health Organization/International Society of Hypertension (WHO/ISH) risk prediction charts, and the appropriate intervention and interval between visits were scheduled based on the level of risk (16, 17). The current study examined the compliance of individuals with the scheduled visits, i.e., whether they visited at the scheduled time or not. We also examined the factors associated with this compliance.

## 2. Method

### 2.1. Study population

The IraPEN program has been derived from the WHO-PEN program and matched with existing national programs for non-communicable diseases (NCDs). We used data from the Ministry of Health's IraPEN Program in the pilot phase during 2016–2018 in four cities: Naghadeh, Maragheh, Shahreza, and Baft. Maragheh is a city in the East Azerbaijan province, north of Iran; Naghadeh is a city in the West Azerbaijan province, northwest of Iran, located near the border with Turkey; Baft is a city in the Kerman province, south of Iran; and Shahreza is a city in the Isfahan province, center of Iran.

In IraPEN, individuals aged 30 years and older were called to the health center in their area for screening for cardiovascular risk factors. The calculation of 10-year CVD risk was applied to those who were 40 years old and over or 30–39 years old with at least one risk factor for cardiovascular disease. The WHO/ISH risk assessment chart for the Eastern Mediterranean Region B was applied using features of smoking status, diabetes, systolic blood pressure, total cholesterol, and age. According to this chart (18), people were placed in four risk groups as follows:

- Low risk (<10%): care and risk assessment were followed annually.
- Medium risk (10– <20%): care and risk assessment were followed every 9 months.
- High risk (20– <30%): care and risk assessment were followed every 6 months.
- Very-high risk (30% and above): care and risk assessment were followed every 3 months.

After reviewing the initial data of 166,158 participants aged 30 years and over, individuals with a history of myocardial infarction, positive angiography, coronary artery bypass grafting, stroke, or deep vein thrombosis (4,921 people) were excluded. We also excluded 404 participants under 30 years of age who were wrongly admitted to the program, and the remaining 160,833 subjects were included in the statistical analysis. Among them, 24,264 participants aged 30–39 years who had no CVD risk factor were not included in the risk scoring.

### 2.2. Data collection

All related data, including personal characteristics, BMI, history of cardiovascular disease, diabetes, high blood pressure, history of smoking or alcohol consumption, and family history of premature cardiovascular disease and diabetes, were collected, and blood pressure, waist circumference, blood sugar, and blood cholesterol were measured. Point-of-care instruments (LipidPro tester) were used for blood sugar and cholesterol measurements. Then, appropriate interventions, including lifestyle modification, drug treatment, and/or referral to the secondary prevention level, were considered according to the individuals' risk factors and level of 10-year CVD risk (19).

In this study, we calculated the rate of compliance with the referral schedule by checking the referral times of individuals according to their 10-year CVD risk. Compliance was defined as adherence to the referral schedule, rather than compliance with prescribed medications or behavioral changes. To evaluate this compliance, any visit within the range of 50% to 150% of the time scheduled was considered desirable, and a visit outside of this range was undesirable. We also categorized visits before 50% of the time as an early referral and visits after 150% of the time as a late referral. Total compliance was defined as any compliance (desirable, early, and late compliance) vs. non-compliance (19).

Supplementary Table 1 shows the definition and further details of compliance based on the referral times at different levels of 10-year CVD risk.

### 2.3. Statistical analysis

A descriptive analysis of the data on variables was carried out for the total sample (n=160,833), reporting the mean (standard deviation) for age and BMI and relative frequencies for categorical variables. The *t*-test and chi-square tests were applied to quantitative and qualitative variables, respectively.

Logistic regression models examined associations between total compliance as a binary variable, i.e., any compliance (desirable, early, and late compliance) vs. non-compliance and proposed covariates including age and sex, diabetes, hypertension, hypercholesterolemia, family history of diabetes, family history of premature CVD, smoking as the binary variables, BMI, and the city of residence as the categorical variables in a stepwise manner (with entering and removal probabilities of 0.1); all variables were finally selected. Interaction between sex and other variables was investigated; some interaction terms were significant (age, BMI range 18– <25, family history of diabetes, family history of premature CVD), so we ran separate logistic regression models by sex for all covariates. To evaluate collinearity in the regression models, Pearson's correlation matrices for all variables were calculated. In addition, to evaluate multicollinearity, the variance inflation factor (VIF) was used to assess the extent to which the variances of the estimated coefficients were inflated. A variable with a VIF >10 was considered an indication of serious collinearity. To explore the association between compliance and the level of CVD risk, logistic regression was used. Model 1 was univariable and ran for crude odds ratio, model 2 was adjusted for age, and model 3 was further adjusted for all covariates considered in the previous multivariable logistic regression.

## 3. Results

We studied 93,423 women and 67,410 men with a mean (SD) age of 46.9 (12.9) and 47.7 (13.6) years, respectively. Table 1 shows the baseline characteristics of the participants.

**Table 1:** Baseline characteristics of participants in the IraPEN pilot program.

**Characteristic**		**Total (160,833)**	**Female (93,423)**	**Male (67,410)**
			**N (%)**	
**Risk score**				
	<10%	116,469 (95.5)	66,396(95.4)	50,073(95.6)
	10%– <20%	4,382 (3.6)	2,500(3.6)	1,882(3.6)
	20%– <30%	939 (0.8)	607(0.9)	332(0.7)
	≥30%	174 (0.1)	102(0.1)	72(0.1)
**Age**				
	Mean (SD)	47.26 (13.3)	46.93(12.99)	47.75(13.66)
	30–39.99	56,263 (35.0)	33,372(35.7)	22,891(34.0)
	40–59.99	74,827 (46.5)	43,405(46.5)	31,422(46.6)
	≥60	29,743 (18.5)	16,646(17.8)	13,097(19.4)
**BMI**				
	Mean (SD)	27.23 (4.93)	28.29(5.14)	25.76(4.19)
	Underweight (<18)	2,124 (1.3)	896(1.0)	1,228(1.8)
	Normal (18– <25)	55,101 (34.3)	25,019(26.8)	30,082(44.7)
	Overweight (25– <30)	60,826 (37.9)	35,008(37.5)	25,818(38.4)
	Obese (30 or higher)	42,509 (26.5)	32,350(34.7)	10,159(15.1)
**Smoking**				
		9,407(5.9)	530 (0.6)	8,877(13.2)
**HTN**				
		25,796(16.0)	17,729 (19.0)	8,067(12.0)
**Diabetes**				
		15,208(9.5)	10,323 (11.0)	4,885(7.3)
**Hypercholesterolemia**				
		22,206(13.8)	15,478 (16.6)	6,728(10.0)
**Family history of diabetes**				
		23,147(14.4)	14,626 (15.7)	8,521(12.6)
**Family history of premature CVD**				
		17,633 (11.0)	11,024 (11.8)	6,609 (9.8)

All differences between sex were statistically significant (P < 0.001).

The highest rate of total compliance (desirable compliance plus early and late compliance) was observed in moderate-to-high-risk groups (ranging from 31 to 35% in men and 37 to 41% in women). Desirable compliance was higher in those with risks lower than 20%, defined as low to moderate risk (~12% in men and 17% in women). Desirable compliance and total compliance were 13.5% and 20.4%, respectively (Table 2).

**Table 2:** Absolute and relative frequency of desirable and total “compliance to revisit” by risk and sex.

	**Risk**	**Desirable compliance**			**Total compliance**		
		**Male**	**Female**	**Both male and female**	**Male**	**Female**	**Both male and female**
		**N (%) CI**	**N (%) CI**		**N (%) CI**	**N (%) CI**	
1	<10%	6,062 (12.1) 11.8–12.4	11,689 (17.6) 17.3–17.9	17,751 (15.2) 15.03–15.44	8,834 (17.6) 17.3–18	17,115 (25.8) 25.4–26.1	25,949 (22.3) 22.0–22.5
2	10%– <20%	234 (12.4) 10.9–13.9	423 (16.9) 15.5–18.3	657 (14.9) 13.96–16.08	593 (31.5) 29.4–33.6	938 (37.5) 35.6–39.4	1,531 (34.9) 33.5–36.3
3	20%– <30%	16 (4.8) 2.5–7.1	46 (7.6) 5.5–9.7	62 (6.6) 5.18–8.38	115 (34.6) 29.5–39.8	247 (40.7) 36.8–44.6	362 (38.5) 35.4–41.7
4	≥30%	4 (5.6) 0.2–10.9	7 (6.9) 1.9–11.8	11 (6.3) 3.52–11.07	11 (15.3) 7–23.7	27 (26.5) 17.9–35	38 (21.8) 16.3–28.6
	Total	6,316 (10.9) 10.7–11.2	12,165 (15.4) 15.2–15.6	18,481 (13.5) 13.3–13.7	9,553 (16.5) 16–16.9	18,327 (23.3) 23–23.6	27,880 (20.4) 20.2–20.6

Table 3 contains the results of univariable, multivariable, and sex-stratified logistic regression models for related factors to compliance. Based on Pearson's correlation matrices, there was no significant correlation between any two independent variables. The mean VIF was <2, and no variable had a VIF above 10. Several factors were significantly related to compliance. Compared to men, women were 50% more likely to refer to the health center for revisiting. Each 5 year increase in age increased the odds of compliance by 10%, and the main cardiovascular risk factors, including diabetes, hypertension, and hypercholesterolemia, were accompanied by a decline of 30–50%. Obesity and overweight were not associated with compliance except for obesity in men, which reduced compliance slightly (OR = 0.92, 95% CI 0.86–0.99). A family history of diabetes and premature CVD increased the chance of revisiting by 10–30%. All these associations did not change dramatically in multivariable analysis. Residence made a difference in compliance. People in Naghadeh, and somehow in Maragheh, had lower compliance, especially women.

**Table 3:** Logistic regression for associated factors to “compliance to revisits”.

**Variables**	**Crude model**	**Multivariable model**	**Sex-stratified multivariable model**	
			**Male**	**Female**
**Sex**				
**Male**	Reference			
**Female**	1.52 (1.48–1.57)	1.41 (1.36–1.45)		
Age^*^ (for increase every 5 years)				
	1.09 (1.08–1.09)	1.06 (1.05–1.07)	1.08 (1.07–1.09)	1.05 (1.04–1.06)
BMI	
Normal^*^ (18 to <25)	Reference			
Underweight (<18)	0.87 (0.76–0.99)	0.84 (0.73–0.96)	0.72 (0.60–0.87)	1.01 (0.83–1.22)
overweight (25 to <30)	1.04 (1.00–1.07)	1.00 (0.97–1.04)	0.98 (0.94–1.04)	1.02 (0.98–1.07)
Obese (30 or higher)	1.11 (1.07–1.15)	0.98 (0.94–1.01)	0.92 (0.86–0.99)	1.00 (0.96–1.06)
Diabetes				
No	Reference			
Yes	0.56 (0.54–0.58)	0.74 (0.71–0.77)	0.76 (0.71–0.82)	0.72 (0.69–0.76)
Hypertension				
No	Reference			
Yes	0.49 (0.47–0.50)	0.63 (0.61–0.65)	0.65 (0.62–0.70)	0.62 (0.59–0.65)
Hypercholesterolemia			
No	Reference			
Yes	0.69 (0.67–0.72)	0.78 (0.76–0.81)	0.82 (0.77–0.88)	0.77 (0.74–0.81)
Smoking				
No	Reference			
Yes	0.70 (0.66–0.74)	0.91 (0.86–0.97)	0.91 (0.86–98)	0.89 (0.73–1.09)
Family history of diabetes^*^			
No	Reference			
Yes	1.13 (1.09–1.17)	1.09 (1.05–1.13)	1.15 (1.08–1.23)	1.06 (1.02–1.11)
Family history of premature CVD^*^			
No	Reference			
Yes	1.25 (1.20–1.30)	1.28 (1.23–1.34)	1.40 (1.32–1.51)	1.22 (1.16–1.28)
Residence				
Shahreza	Reference			
Maragheh	0.93 (0.90–0.96)	0.91 (0.88–0.94)	0.98 (0.93–1.03)	0.86 (0.83–0.90)
Naghadeh	0.68 (0.66–0.71)	0.65 (0.62–0.68)	0.82 (0.77–0.88)	0.56 (0.53–0.59)
Baft	1.02 (0.97–1.07)	0.98 (0.94–1.03)	1.05 (0.97–1.15)	0.94 (0.89–1.00)

* Interaction of these variables with sex was significant.

Table 4 contains the results of univariable, age-adjusted, and multivariable logistic models for levels of CVD risk by sex. Individuals with moderate-to-high risk of CVD had higher compliance with the program compared to low-risk ones, and very-high-risk subjects were the same as the low risks. However, in the multivariable model containing CVD risk factors, all moderate- to very-high-risk groups had lower compliance to revisit.

**Table 4:** Logistic regression to compare the “compliance to revisit” in different categories of CVD risk.

**Risk score**	**Univariable model**	**Age-adjusted model**	**Multivariable model**
Male			
Risk <10%	Reference		
Risk 10–20%	2.14 (1.94–2.37)	1.23 (1.11–1.37)	0.92 (0.82–1.03)
Risk 20–30%	2.47 (1.97–3.10)	1.44 (1.14–1.82)	0.88 (0.69–1.12)
Risk >30%	0.84 (0.44–1.60)	0.49 (0.26–0.95)	0.29 (0.15–0.57)
Female			
Risk <10%	Reference		
Risk 10–20%	1.73 (1.59–1.87)	1.05 (0.95–1.14)	0.77 (0.70–0.84)
Risk 20–30%	1.97 (1.67–2.32)	1.20 (1.02–1.42)	0.78 (0.65–0.92)
Risk >30%	1.03 (0.66–1.60)	0.63 (0.41–0.99)	0.35 (0.22–0.55)

Multivariable model includes age, diabetes, hypertension, hypercholesterolemia, smoking, family history of premature CVD, family history of diabetes, BMI, and residence.

## 4. Discussion

One of the challenges to implementing preventive measures and disease prevention, in addition to the lack of facilities, is non-compliance. We evaluated the value of this problem and its associated factors in a large sample of people who were the target of preventive healthcare programs. The compliance rate with timely referral for scheduled preventive interventions was very low and related to the individuals' baseline risk factors, and in men, it was worse than in women.

Yang et al. showed that the amount of treatment compliance is related to the risk of CVD. There was a higher risk of stroke in people with low compliance (<40%) compared with people with good compliance (~80%). People who comply 40–79% have a moderate risk of cardiovascular disease (20). In this study, almost two-thirds of the patients were completely lost to the system, and the total compliance rate in the present study was 20.4%. This low rate can be due to the newness of the program and its unfamiliarity to the people under research. Lack of understanding of the benefits of medication, treatment, and recommendations (21), as well as a lack of facilities and insufficient social and family support, can all contribute to low compliance (22). Similar to our study, Martinez et al., who reviewed the World Health Organization's essential non-communicable disease package, implemented only 44% of PEN in full. Despite providing all equipment and training for all service providers, they reached more than half of their total target, which was 80% (23). The vast majority of the population in our study was in the lowest-risk category. It seems that low-risk individuals are not motivated to seek medical care. Furthermore, as shown in a review by Karmali et al., although compliance is one of the secondary consequences of CVD risk scoring and can improve the behavior of the subjects, there is no certainty for higher compliance after risk scoring compared to conventional care (24).

In the study of Fernandez-Lazaro et al., the proportion of patients who complied with the treatments (according to the Morisky Green Levine questionnaire) was 55.5%. Approximately 44.5% of people had poor compliance. One of the main reasons for people's lack of compliance was forgetting to take medication (79%) (25). As reported in previous studies, low compliance is a main and costly obstacle in the management of prevalent non-communicable chronic diseases, such as diabetes and hypertension (26). In a study in Jordan on Syrian refugee patients, they were unwilling to start, stop, or change medication and were less able to improve risk factors and use health education because of their social and economic status (27).

A study in the US in 2010 showed that women were less likely to comply with the use of chronic medications compared to men and were less likely to receive medication and monitoring based on clinical guidelines (28). Although we did not assess medication compliance directly, our findings are opposite to these results, and we showed that Iranian women are approximately 50% more likely than men to be compliant with the scheduled visits recommended by WHO guidelines for CVD prevention. In a previous study of patients with type 1 diabetes, compliance to pharmacological intervention and prescription of statins and aspirin in men was higher than in women (29). In another study, the results showed that there were no differences in sex between medium-high-compliant and low-compliant subjects. The sex-stratified analysis demonstrated that low-compliant men were more likely to be employed (30). In another study, no significant sex differences were observed (31, 32). The results of the Halt et al.'s study revealed that the prevalence of low medication compliance scores did not vary by sex, but factors related to low antihypertensive medication compliance scores are different in men and women (33). Some studies revealed low antihypertensive medication compliance in older women compared to men (34). In a study by Hazuda et al., not only men did not refer for treatment until the advanced stages of the disease but also they had low compliance when prescribed in the advanced stages of treatment (35).

In Raparelli et al.'s study, low-compliant patients were more likely to be diabetic as compared with compliant patients (30), and in a study by Kennedy-Martin et al., drug compliance in patients with type 2 diabetes was associated with lower healthcare costs, reduced hospitalization costs, improved disease control, and reduced complications (36). In our study also, compliance among people who had diabetes was lower than that of those without diabetes.

Literature reviews show that interactions between a family history of diabetes and sex and compliance with regular exercise were significant. Individuals who had a family history of diabetes were more likely to comply with a regular exercise regimen (37). A family history of the disease can induce an understanding of the risk of the disease (38), and people with a family history of diabetes try to reduce or control the risk of family history by changing and improving their lifestyle and healthy behaviors (39).

In a study by Imes et al., there was a positive relationship between reported heart disease in the family and perceived risk, but there was a somewhat conflicting relationship between family history and changes in health-related behavior in relatives; on the other hand, family history of CVD may improve knowledge but not attitude and practice. They concluded that knowledge of the degree of illness in relatives or the risk of heart disease in them was not a sufficient predictor of behavior change in people with a family history of diabetes (40). One of the reasons for low compliance in people with a family history of disease is mentioned in the Allen et al.'s study. Although participants had several modifiable risk factors and a family history of coronary artery disease, they did not accept being at risk for the disease, and their perceived risk for heart disease was low (41).

We understand that compliance is significantly related to age. In this study, older individuals were more compliant than their young counterparts (approximately a 10% increase per every 5-year increase in age). In Fernandez-Lazaro et al.'s study, similarly older age was related to compliance (30% increase per 10-year increase in age) (25). In a study by Aljabri et al., compliance with the Mediterranean diet increased with age, which was in line with our study (42). One of the reasons for the higher compliance of people of older ages can be due to the free time of this group of clients. Older people are better able to go to the centers due to retirement and not being at work during the day, and consequently, the rate of compliance is higher.

Smoking, one of the high-risk habits, is a known risk factor for CVD (43). Smokers complied less with the programs in our study, but this association decreased after adjustment for other risk factors. In a study by Raparelli et al., in an age and sex-adjusted analysis, active smoking was independently associated with low Mediterranean diet compliance (44). Active smoking was negatively associated with compliance with anti-tuberculosis treatment (45). Furthermore, in another study, current smokers were less likely to comply with their treatment by 7% for diabetes and 9% for hypertension (46).

In our study, being underweight and obese were negative predictors of compliance just in men. In a study by Warren et al., obesity was a negative predictor of compliance with treatment (47). A prospective study reported that being overweight or obese is among the negative predictors of blood pressure control (48).

In this study, compliance was operationalized and defined as compliance to referral, not compliance to taking prescribed medications or behavioral lifestyle changes, and the overall compliance rate was 20.4%. However, one of the goals of this program was risk assessment and then increasing the attention and follow-up of people at risk of cardiovascular disease for consequent behavior change, smoking cessation, and reducing high blood pressure and cholesterol. There is no assurance that increased risk awareness in individuals will lead to increased compliance with revisits. After adjustment for risk factors, the higher risks did not increase compliance with the program anymore and even decreased compliance, and it seems that individuals follow the program just for their risk factors such as diabetes and hypertension. Given the low compliance in this study, it is not clear whether the low compliance to revisit is related to the low attention of people to the first-level preventive care program or whether they refer to a private office and do not go to health centers.

This study was performed on the national data of the IraPEN pilot program, which has been derived from the comprehensive WHO-PEN program. In addition to its strengths, the study also had some limitations. In this study, we did not measure compliance with any medication or behavioral lifestyle modification recommended by health professionals. One of the most important limitations was conducting a study in the pilot phase of the program, which was faced with low stability of results due to the short period of the pilot phase. The pilot nature of the program and its novelty reinforced the lack of compliance among people with different economic and social levels. Furthermore, the high sample size made any difference significant, which may or may not be related to the outcome of interest. In this regard, we just considered the effect sizes above 10% and ignored the values lower than 10% in our conclusion. Another limitation of the study is the lack of access to all demographic and socioeconomic variables, such as occupation and education, for analysis. In our study, it was not clear whether the lack of follow-up was due to a lack of attention to prevention programs, a lack of trust in health centers, a lack of access to health centers, or patients' referral to private centers. Further research is also needed to determine adherence to medication and lifestyle modification. It seems that most individuals have high knowledge and attitudes toward CVD, but their practice is not well (49). Therefore, policymakers should spend more on the education of individuals about CVD risk factors and increase people's sensitivity to seeking and following medical care, especially preventive care.

## 5. Conclusion

In this study, the compliance rate was low, which could be due to people's unfamiliarity with the IraPEN program. According to the results of logistic regression, factors such as hypertension, diabetes, and hypercholesterolemia were negatively associated with revisit compliance, while family history of diabetes and premature CVD were positively associated. Independent of these factors, people with higher calculated risk showed lower compliance. There were a few interactions between sex and other factors for compliance with revisits for preventive strategies. Careful planning to increase compliance among people with cardiovascular risk factors is recommended.

## Data availability statement

The original contributions presented in the study are included in the article/Supplementary material, further inquiries can be directed to the corresponding author/s.

## Ethics statement

Ethical Approval was granted from the Ethics Committee and Review Board of the School of Public Health and Safety, Shahid Beheshti University of Medical Sciences (SBMU), Tehran, Iran (IR.SBMU.PHNS.REC.1397.086). Informed consent was obtained from all participants.

## Author contributions

DK and S-SH-N developed the conceptual framework and verified the analytical methods. FH performed the analysis and wrote the first draft and edited the final draft with DK. S-SH-N and AO supervised the project, interpreted the results, and revised the paper carefully. AM, MA, and SD reviewed and revised the paper carefully.
