# Supplementary material for: Timely referral to health centers for the prevention of cardiovascular diseases: IraPEN national program
Source: Front Public Health. 2023 Sep 22;11:1098312. doi: 10.3389/fpubh.2023.1098312 (PMC10556464; doi:10.3389/fpubh.2023.1098312)
Supplement: Supplementary file 1 [file Table_1.docx]

Supplementary table 1: Definition of desirable, early, and late referral, as different levels of compliance to revisit in low-, moderate-, high-, and very high-risk participants regarding cardiovascular diseases

|  | Low risk  (Less than 10%) | Moderate risk  (10 to less than 20%) | High risk  (20 to less than 30%) | Very High risk )≥30%( |
| --- | --- | --- | --- | --- |
| Recommendation | Timely referral 1 year later | Timely referral 9 months later | Timely referral 6 months later | Timely referral 3 months later |
| Level of compliance |  | | | |
| Early referral | Revisit before 182 days later | Revisit before 135 days later | Revisit before 90 days later | Revisit before 45 days later |
| Desirable referral | Revisit between 182 days to 548 days later | Revisit between 135 days to 405 days later | Revisit between 90 days to 270 days later | Revisit between 45 days to 135 days later |
| Late referral | Revisit after 548 days | Revisit after 405 days | Revisit after 270 days | Revisit after 135 days |
